# Alterations of Ultra Long-Chain Fatty Acids in Hereditary Skin Diseases—Review Article

**DOI:** 10.3389/fmed.2021.730855

**Published:** 2021-08-23

**Authors:** Agata Zwara, Katarzyna Wertheim-Tysarowska, Adriana Mika

**Affiliations:** ^1^Department of Environmental Analysis, Faculty of Chemistry, University of Gdansk, Gdansk, Poland; ^2^Department of Medical Genetics, Institute of Mother and Child, Warsaw, Poland; ^3^Department of Pharmaceutical Biochemistry, Faculty of Pharmacy, Medical University of Gdansk, Gdansk, Poland

**Keywords:** lipids, fatty acids, skin, epidermis, cholesterol, ceramides, dermis

## Abstract

The skin is a flexible organ that forms a barrier between the environment and the body's interior; it is involved in the immune response, in protection and regulation, and is a dynamic environment in which skin lipids play an important role in maintaining homeostasis. The different layers of the skin differ in both the composition and amount of lipids. The epidermis displays the best characteristics in this respect. The main lipids in this layer are cholesterol, fatty acids (FAs) and ceramides. FAs can occur in free form and as components of complex molecules. The most poorly characterized FAs are very long-chain fatty acids (VLCFAs) and ultra long-chain fatty acids (ULCFAs). VLCFAs and ULCFAs are among the main components of ceramides and are part of the free fatty acid (FFA) fraction. They are most abundant in the brain, liver, kidneys, and skin. VLCFAs and ULCFAs are responsible for the rigidity and impermeability of membranes, forming the mechanically and chemically strong outer layer of cell membranes. Any changes in the composition and length of the carbon chains of FAs result in a change in their melting point and therefore a change in membrane permeability. One of the factors causing a decrease in the amount of VLCFAs and ULCFAs is an improper diet. Another much more important factor is mutations in the genes which code proteins involved in the metabolism of VLCFAs and ULCFAs—regarding their elongation, their attachment to ceramides and their transformation. These mutations have their clinical consequences in the form of inborn errors in metabolism and neurodegenerative disorders, among others. Some of them are accompanied by skin symptoms such as ichthyosis and ichthyosiform erythroderma. In the following review, the structure of the skin is briefly characterized and the most important lipid components of the skin are presented. The focus is also on providing an overview of selected proteins involved in the metabolism of VLCFAs and ULCFAs in the skin.

## Introduction

The skin is a large organ composed of three main layers: hypodermis, dermis, and epidermis. The primary role of the hypodermis is protection against mechanical injury, and thermal insulation. In addition, it provides support and energy for the body [fat cells store triacylglycerols (TAGs), which are produced during lipogenesis] (1, 2). The dermis is involved in the body's immune defense; it provides elasticity and moisture to the skin (3), and epidermal nourishing and support (1, 3, 4). The dermal-epidermal junction (DEJ) is the connection between the dermis and the epidermis. The DEJ includes complex junctional structures in the dermo-epidermal junction areas. The role of the DEJ is to assist in the adhesion of the epidermis to the dermis and to regulate the exchange of metabolic products. It also plays a role in the migration of keratinocytes during the wound-healing process (1, 3, 4). The outermost layer of the skin, being the actual physical barrier between the body and the environment, is the epidermis.

Among the most important components of human skin are lipids. These hydrophobic molecules are important for the proper functioning of the protective barrier—they prevent the entry of microorganisms and inhibit transepidermal water loss (TEWL).

In the skin, the most abundant lipids are cholesterol, free fatty acids (FFAs) and ceramides (CERs). Very long-chain fatty acids (VLCFAs) and ultra long-chain fatty acids (ULCFAs) are part of the FFA fraction, and major components of ceramides. VLCFAs have chain lengths of 20–25 carbon atoms. FAs which have 26 or more carbon atoms in their chains are called ULCFAs (5, 6). VLCFAs and ULCFAs are responsible for the rigidity and impermeability of membranes, forming the mechanically and chemically robust outermost layer of cell membranes. Any change in the composition and length of the carbon chains of fatty acids (FAs) results in changes in their melting points. Despite playing such an important role, the number of papers concerning VLCFAs and ULCFAs in different tissues is highly limited.

### Lipid Composition in Human Skin

The composition of lipids differs in each part of the skin. In the hypodermis two main lipid groups, TAGs and FFAs, can be distinguished (Table 1). In the dermis, which is rich in collagen and elastin fibers, high concentrations of TAGs and diacylglycerols (DAGs) are localized in deep areas (Table 1). There are also eight classes of ceramides with the predominance of a non-hydroxy FA chain, as well as eleven subtypes of phospholipids (23) (Table 1). The lipid content in the epidermis is much more complex, as was described above.

**Table 1:** The composition of skin lipids in particular skin layers.

**Layer of skin**		**Lipids**	**Individual species**	**Number of studied subjects**	**Age**	**Sex**	**References**
Epidermis	Stratum corneum	Cholesterol esters	nd	22	22–40 y	F	Norlen et al. (7)
		TAG	nd	4 cadavers	nd	nd	Lampe et al. (8)
		FFA from abdomen	C14:0 (3.8%), C16:0 (36.8%), C16:1 (3.6%), C18:0 (9.9%), C18:1 (33.1%), C18:2 (12.5%), C20:0 (0.3%), C20:1 (trace), C22:0 (trace)	nd	Median age of 50 y	M	Lampe et al. (9)
		FFA from leg	C14:0 (10.9%), C16:0 (36.2%), C16:1 (16.6%), C18:0 (10.0%), C18:1 (17.7%), C18:2 (1.4%), C20:0 (2.6%), C20:1 (1.1%), C20:2 (trace), C20:3 (trace), C20:4 (trace), C22:0 (3.5%)	nd	Median age of 50 y	M	Lampe et al. (9)
		FFA from plantar	C14:0 (0.3%), C16:0 (10.5%), C16:1 (1.2%), C18:0 (20.1%), C18:1 (18.8%), C18:2 (6.5%), C20:0 (6.1%), C20:1 (1.5%), C20:3 (3.1%), C22:0 (9.6%), C22:1 (5.8%), C24:0 (16.5%)	nd	nd	M	Lampe et al. (9)
		FFA from face	C14:0 (1.4%), C16:0 (27.9%), C16:1 (6.5%), C18:0 (16.3%), C18:1 (23.5%), C18:2 (11.9%), C20:0 (2.4%), C20:1 (0.1%), C20:2 (0.1%), C20:4 (3.5%), C22:0 (4.4), C22:1 (2.0%)	nd	nd	M	Lampe et al. (9)
		FFA from forearm	^**^C12:0, C14:0, C16:0, C16:1, C18:0, C19:0, C20:0, C21:0, C22:0, C24:0, C25:0, C26:0, C27:0, C28:0, C29:0, C30:0, C30:1, C31:0, C32:0, C32:1, C34:0, C34:1, C36:0, C36:1	22	22–40 y	F	Norlén et al. (7)
		FFA from stripped sample from forearm	C20:0 (5%), C22:0 (11%), C24:0 (39%), C25:0 (10%), C26:0 (23%), C27:0 (3%), C28:0 (8%), C29:0 (1%), C30:0 (2%)	22	22–40 y	F	Norlén et al. (7)
		FA in SC ceramide Cer[NS] from forearm^C^	C24:0 (8.95%), C25:0 (6.97%), C26:0 (10.77%), C27:0 (5.16%), C28:0 (11.99%), C29:0 (5.92%), C30:0 12.59%), C31:0 (7.13%), C32:0 (14.87%), C33:0 (5.77%), C34:0 (10.77%)	7	37 ± 13 y	5 F 2 M	Farwanah et al. (10)
		FA in SC ceramide Cer[NDS] from forearm^C^	C24:0 (6.50%), C25:0 (4.72%), C26:0 (13.19%), C27:0 (8.27%), C28:0 (19.69%), C29:0 (9.65%), C30:0 (18.31%), C31:0 (7.48%), C32:0 (12.20%)	7	37 ± 13 y	5 F 2 M	Farwanah et al. (10)
		FA in SC ceramide Cer[NP] from forearm^C^	C24:0 (9.78%), C25:0 (6.99%), C26:0 (13.17%), C27:0 (7.98%), C28:0 (19.96%), C29:0 (9.98%), C30:0 (17.76%), C31:0 (5.99%), C32:0 (8.38%)	7	37 ± 13 y	5 F 2 M	Farwanah et al. (10)
		FA in SC ceramide Cer[NH] from forearm^C^	C24:0 (7.28%), C25:0 (10.24%), C26:0 (26.95%), C27:0 (10.51%), C28:0 (20.22%), C29:0 (7.55%), C30:0 (17.25%)	7	37 ± 13 y	5 F 2 M	Farwanah et al. (10)
		FA in SC ceramide Cer[AS] from forearm^C^	C15:0 (17.37%), C16:0 (52.63%), C17:0 (11.58%), C18:0 (18.42%)	7	37 ± 13 y	5 F 2 M	Farwanah et al. (10)
		FA in SC ceramide Cer[AP] from forearm^C^	C24:0 (21.08%), C25:0 (11.48%), C26:0 (19.91%), C27:0 (10.54%), C28:0 (21.78%), C29:0 (7.49%), C30:0 (7.73%)	7	37 ± 13 y	5 F 2 M	Farwanah et al. (10)
		FA in SC ceramide Cer[AH] from forearm^C^	C24:0 (21.07%), C25:0 (14.64%), C26:0 (35.71%), C27:0 (10.71%), C28:0 (17. 86%)	7	37 ± 13 y	5 F 2 M	Farwanah et al. (10)
		FA in SC ceramide Cer[EOS] from forearm^C^	C30:0 (6.82%), C31:0 (5.80%), C32:0 (18.77%), C33:0 (11.26%), C34:0 (34.13%), C35:0 (10.92%), C36:0 (12.29%)	7	37 ± 13 y	5 F 2 M	Farwanah et al. (10)
		FA in SC ceramide Cer[EOP] from forearm^C^	C30:0 (13.05%), C31:0 (5.93%), C32:0 (18.10%), C33:0 (13.06%), C34:0 (29.67%), C35:0 (10.09%), C36:0 (10.09%)	7	37 ± 13 y	5 F 2 M	Farwanah et al. (10)
		FA in SC ceramide Cer[EOH] from forearm^C^	C30:0 (24.18%), C31:0 (12.82%), C32:0 (36.63%), C33:0 (11.36%), C34:0 (15.02%)	7	37 ± 13 y	5 F 2 M	Farwanah et al. (10)
		FA in SC ceramide Cer[NS] from forearm	^**^C16:0, C17:0, C18:0, C19:0, C20:0, C21:0, C22:0, C23:0, C24:0, C25:0, C26:0, C27:0, C28:0, C29:0, C30:0, C30:1	19	20–50 y	9 F 10 M	Kawana et al. (11)
		FA in SC ceramide Cer[NDS] from forearm	^**^C16:0, C17:0, C18:0, C19:0, C20:0, C21:0, C22:0, C23:0, C24:0, C25:0, C26:0, C27:0, C28:0, C29:0, C30:0, C30:1	19	20–50 y	9 F 10 M	Kawana et al. (11)
		FA in SC ceramide Cer[NH] from forearm	^**^C16:0, C17:0, C18:0, C20:0, C21:0, C22:0, C23:0, C24:0, C25:0, C26:0, C27:0, C28:0, C29:0, C30:0, C30:1	19	20–50 y	9 F 10 M	Kawana et al. (11)
		FA in SC ceramide Cer[NP] from forearm	^**^C16:0, C20:0, C22:0, C23:0, C24:0, C25:0, C26:0, C27:0, C28:0, C29:0, C30:0, C30:1	19	20–50 y	9 F 10 M	Kawana et al. (11)
		FA in SC ceramide Cer[AS] from forearm	^**^C16:0, C17:0, C18:0, C20:0, C22:0, C23:0, C24:0, C25:0, C26:0, C27:0, C28:0, C30:0	19	20–50 y	9 F 10 M	Kawana et al. (11)
		FA in SC ceramide Cer[AH] from forearm	^**^C16:0, C17:0, C18:0, C20:0, C22:0, C23:0, C24:0, C25:0, C26:0, C27:0, C28:0, C30:0	19	20–50 y	9 F 10 M	Kawana et al. (11)
		FA in SC ceramide Cer[AP] from forearm	^**^C16:0, C17:0, C18:0, C19:0, C20:0, C21:0, C22:0, C23:0, C24:0, C25:0, C26:0, C27:0, C28:0, C29:0, C30:0	19	20–50 y	9 F 10 M	Kawana et al. (11)
		FA in SC ceramide Cer[EOS] from forearm	^**^C28:0, C29:0, C30:0, C31:0, C32:0, C32:1, C33:0, C33:1, C34:0, C34:1, C36:1	19	20-50y	9 F 10 M	Kawana et al. (11)
		FA in SC ceramide Cer[EOH] from forearm	^**^C28:0, C29:0, C30:0, C31:0, C32:0, C32:1, C33:0, C33:1, C34:0, C34:1, C36:1	19	20–50 y	9 F 10 M	Kawana et al. (11)
		FA in SC ceramide Cer[EOP] from forearm	^**^C28:0, C29:0, C30:0, C31:0, C32:0, C32:1, C33:0, C34:0, C34:1, C36:1	19	20–50 y	9 F 10 M	Kawana et al. (11)
		FA in SC ceramides from abdomen	C16:0 (7.7%), C18:0 (4.8%), C18:1 (6.3%), C18:2 (14.0%), C20:0 (5.9%), C24:0 (50.8%), C26:0 (10.5%)	nd	Median age of 50 y	M	Lampe et al. (9)
		FA in SC ceramides from leg	C16:0 (10.2%), C18:0 (11.4%), C18:1 (3.6%), C18:2 (1.9%), C24:0 (43.3%), C26:0 (29.6%)	nd	Median age of 50 y	M	Lampe et al. (9)
		FA in SC ceramides from face	C14:0 (0.1%), C16:0 (4.3%), C18:0 (9.8%), C18:1 (4.3%), C18:2 (6.1%), C20:0 (3.8%), C20:4 (0.3%), C22:0 (7.0%), C22:1 (2.0%), C24:0 (43.9%), C24:1 (10.8%), C26:0 (7.7%)	nd	nd	M	Lampe et al. (9)
		FA in SC wax/sterols from abdomen	C16:0 (20.0%), C16:1 (15.9%), C18:0 (5.8%), C18:1 (49.4%), C18:2 (6.6%), C24:0 (0.9%), C24:1 (1.6%)	nd	Median age of 50 y	M	Lampe et al. (9)
		FA in SC wax/sterols from leg	C14:0 (4.21%), C16:0 (21.0%), C16:1 (27.8%), C18:0 (6.2%), C18:1 (32.9%), C18:2 (5.1%), C20:0 (0.9%), C20:1 (0.7%), C20:2 (trace), C24:0 (1.4%)	nd	Median age of 50 y	M	Lampe et al. (9)
		FA in SC wax/sterols from plantar	C14:0 (2.5%), C16:0 (21.4%), C16:1 (5.7%), C18:0 (8.6%), C18:1 (44.2%), C18:2 (15.2%), C20:1 (trace), C20:4 (trace), C22:1 (trace), C24:0 (2.4%)	nd	nd	M	Lampe et al. (9)
		FA in SC wax/sterols from face	C14:0 (0.9%), C16:0 (14.6%), C16:1 (36.9%), C18:0 (4.6%), C18:1 (32.9%), C18:2 (10.0%), 20:0 (trace). C20:1 (trace), C20:4 (trace), C22:1 (trace)	nd	nd	M	Lampe et al. (9)
		FA in SC phosphatidylethanolamines from abdomen	C14:0 (0.8%), C16:0 (15.8%), C16:1 (4.9%), C18:0 (13.5%), C18:1 (38.1%), C18:2 (20.7%), C20:0 (1.3%), C20:1 (1.0%), C20:2 (0.3%), C20:3 (trace), C20:4 (1.6%), C22:0 (0.7%), C24:1 (1.3%)	nd	Median age of 50 y	M	Lampe et al. (9)
		FA in SC phosphatidylethanolamines from leg	C14:0 (3.0%), C16:0 (10.3%), C16:1 (4.0%), C18:0 (13.6%), C18:1 (34.0%), C18:2 (21.6%), C20:0 (trace), C20:1 (trace), C20:2 (1.2%), C20:3 (trace), C20:4 (12.2%)	nd	Median age of 50 y	M	Lampe et al. (9)
		Total FA in SC from mid-abdominal and mid-scapular	C10:0 (0.7%), C11:0 (0.04%), C12:0 (0.7%), C13:0 (0.2%), C14:0 (4.6%), C14:1 + iso-C14 + anteiso-C14 (0.4%), C16:0 (26.3%), C16:1 + iso-C16 + anteiso-C16 (9.0%), C17:0 (2.2%), C18:0 (3.5%), C18:1 + C18:2 + iso-C18 + anteiso-C18 (52.7%)	17 cadavers	M: 49–68 y F: 2 wks−85 y	8 M 9 F	Reinertson et al. (12)
				9 normal human	23–52 y	M	
		Phospholipids	^**^PE, PS	4 cadavers	nd	nd	Lampe et al. (8)
		Ceramide	Cer [NS] (21.38%), Cer [EOS] (9.45%), Cer [NP] (18.51%), Cer [AS] (25.23%), Cer [AP] (25.43%)	6	nd	F M	Motta et al. (13)
			Cer [NDS] (9.83%), Cer [NT] (1.73%), Cer[NS] (7.44%), Cer [NP] (22.10%), Cer [NH] (14.51%), Cer [AH] (10.77%), Cer [ADS] (1.63%), Cer [AS] (9.58%), Cer [AP] (8.78%), Cer [OH]^a^ (0.43%), Cer [OP]^a^ (0.17%), Cer [OS]^a^ (0.73%), Cer [EOH] (4.26%), Cer [EODS] (0.40%), Cer [EOS] (6.48%), Cer [EOP] (1.14%)	nd	nd	nd	t'Kind et al. (14)
			Cer [NDS] (6.2%), Cer [NS] (5.2%), Cer [NH] (23.7%), Cer [NP] (24.2%), Cer [NSD] (0.1%), Cer [AS] (4.3%), Cer [ADS] (0.9%), Cer [AH] (18.0%), Cer [AP] (9.2%), Cer [ASD] (0.2%), Cer [BS] (0.2%), Cer [OS] (0.6%), Cer [ODS] (0.1%), Cer [OH] (0.6%), Cer [OP] (0.3%), Cer [OSD] (0.02%), Cer [EOS] (2.1%), Cer [EODS] (0.1%), Cer [EOH] (3.1%), Cer [EOP] (1.0%), Cer [EOSD] (0.02%),	19	20–50 y	9 F 10 M	Kawana et al. (11)
			Cer [AH] (22%) Cer [EOS] (8%), Cer [NS] (21%), Cer [NP] (13%), Cer [EOH] (4%), Cer [AS] 27%, Cer [AP] (4%), Cer [OS]^a^ (66%), Cer [OH]^a^ (33%)	nd	26–45 y	M	Robson et al. (15)
			^**^Cer [EODS], Cer [EOS], Cer [EOP], Cer [EOH], Cer[NDS], Cer[NS], Cer [NP], Cer [ADS], Cer [AS], Cer [NH], Cer[AP], Cer [AH]	nd	nd	nd	van Smeden et al. (16)
			^**^Cer [1-O-EAS], Cer [1-O-ENS]	nd	26–45 y	M	Rabionet et al. (17)
			Cer [OS] (72.4%), Cer [OH] (19.5%), Cer [OP] (8.2%)^C^	6	nd	nd	Hill et al. (18)
	Stratum granulosum	TAG (24.7%)	nd	7 cadavers	nd	nd	Lampe et al. (8)
		FFA (9.2%)	nd	7 cadavers	nd	nd	Lampe et al. (8)
		FA in sphingolipids	C14:0 (0.7%), C16:0 (13.1%), C16:1 (1.8%), C18:0 (11.4%), C18:1 (32.3%), C18:2 (18.8%), C20:0 (1.2%), C20:1 (0.4%), C20:4 (1.8%), C22:0 (2.5%), C24:0 (6.8%), C26:0 (9.3%)	nd	nd	nd	Lampe et al. (8)
		FA in neutral lipids	C12:0 (0.3%), C14:0 (3.5%), C16:0 (25.3%), C16:1 (7.4%), C18:0 (16.7%), C18:1 (31.1%), C18:2 (14.3%), C20:0 (0.03%), C20:2 (0.3%), C22:0 (0.4%), C24:0 (0.7%)	nd	nd	nd	Lampe et al. (8)
		FA in phospholipids	C16:0 (9.4%), C18:0 (20.6%), C18:1 (31.0%), C18:2 (26.5%), C20:0 (2.1%), C20:4 (3.6%)	nd	nd	nd	Lampe et al. (8)
		Phospholipids	^**^PC, PE, LCS, PS, PI	7 cadavers	nd	nd	Lampe et al. (8)
		Ceramide	nd	7 cadavers	nd	nd	Lampe et al. (8)
	Stratum spinosum/ Stratum basale	TAG (12,4%)	nd	5 cadavers	nd	nd	Lampe et al. (8)
		FFA (7,0%)	nd	5 cadavers	nd	nd	Lampe et al. (8)
		FA in neutral lipids	C12:0 (0.03%), C14:0 (1.9%), C16:0 (24.1%), C16:1 (6.7%), C18:0 (10.7%), C18:1 (36.8%), C18:2 (14.5%), C20:0 (0.5%), C20:2 (0.5%), C22:0 (0.9%), C24:0 (3.8%)	nd	nd	nd	Lampe et al. (8)
		FA in phospholipids	C16:0 (25.8%), C18:0 (14.1%), C18:1 (42.1%), C18:2 (12.3%)	nd	nd	nd	Lampe et al. (8)
		Total FA in SS/SB from mid-abdominal and mid-scapular	C10:0 (1.9%), C11:0 (0.1%), C12:0 (0.7%), C14:0 (4.2%), C14:1 + iso-C14 + anteiso-C14 (1.0%), C16:0 (25.2%), C16:1 + iso-C16 + anteiso-C16 (5.3%), C18:0 (5.5%), C18:1 + C18:2 + iso-C18 + anteiso-C18 (57.3%)	17 cadavers	M: 49–68 y F: 2 wks−85 y	8 M 9 F	Reinertson et al. (12)
				9 normal human	23–52 y	M	
		Phospholipids	^**^PC, PE, LCS, PS, PI	5 cadavers	nd	nd	Lampe et al. (8)
		Ceramide	nd	5 cadavers	nd	nd	Lampe et al. (8)
	Epidermis^*^	Ceramide	Cer [NS] (34.5%), Cer [NDS](11.7%), Cer [NH] (14.3%), Cer [NP] (12.6%), Cer [AS] (3.3%), Cer [ADS] (1.1%), Cer [AH] (5.2%), Cer [AP] (6.3%), Cer [EOS] (8.8%), Cer [EOH] (1.8%), Cer [EOP] (0.4%)	4	33–47 y	F	Kendall et al. (19)
			Cer [OH]^a^, Cer [OS]^a^, Cer [OT]^a^, Cer [1-O-E(EO)S]^b^, Cer [1-O-E(EO)H]^b^, Cer [1-O-E(EO)T]^b^ – nd	nd	nd	nd	Assi et al. (20)
		FA in sphingomyelin	C14:0 (2.6%), C15:0 (1.1%), C16:0 (14.6%), C17:0 (2.0%), C18:0 (6.4%), C18:1 (2.8%), C20:0 (11.6%), C21:0 (1.3%), C22:0 (8.9%), C23:0 (1.6%), C24:0 (18.8%), C24:1 (9.5%), C25:0 (2.0%), C26:0 (5.8%), C28:0 (0.7%)	nd	nd	nd	Gray and Yardley (21)
		FA of epidermal glycosphingolipid	C14:0 (5.1%), C15:0 (3.4%), C16:0 (8.2%), C17:0 (2.4%), C18:0 (4.3%), C18:1 (17.9%), C20:0 (7.7%), C21:0 (1.7%), C22:0 (4.3%), C23:0 (1.7%), C24:0 (10.0%), C24:1 (2.0%), C25:0 (5.2%), C26:0 (5.4%), C28:0 (5.9%), C24:0-OH (2.6%), C26:0-OH (5.6%)	nd	nd	nd	Gray and Yardley (21)
		FA of prostanoids	C20:3 n-6 (10.2%), C20:4 n-6 (88.3%), C20:5 n-3 (1.5%)	8	28–56 y	F	Kendall et al. (22)
		Hydroxy FA	C18:2 n-6 (69.7%), C20:3 n-6 (1.7%), C20:4 n-6 (25.2%), C20:5 n-3 (2.4%), C22:6 n-3 (1.1%)	8	28–56 y	F	Kendall et al. (22)
		FA of N-acylethanolamides	C16:0 (34.7%), C18:0 (11.4%), C18:1 n-9 (11.3%), C18:2 n-6 (5.5%), C18:3 n-3 (1.2%), C20:4 n-6 (13.1%), C20:5 n-3 (6.3%), C22:6 n-3 (16.5%)	8	28–56 y	F	Kendall et al. (22)
		Total FA	C16:0 (23.9%), C18:0 (22.1%), C18:1 n-9 (24.3%), C18:2 n-6 (9.6%), C18:3 n-3 (0.5%), C20:4 n-6 (2.7%), C20:5 n-3 (0.5%), C22:6 n-3 (0.5%)	8	28–56y	F	Kendall et al. (22)
		Total FA in SC from mid-abdominal and mid-scapular	C10:0 (0.7%), C11:0 (0.1%), C12:0 (0.5%), C13:0 (0.1%), C14:0 (3.6%), C14:1 + iso-C14 + anteiso-C14 (0.5%), C15:0 (1.0%), C16:0 (27.7%), C16:1 + iso-C16 + anteiso-C16 (7.6%), C18:0 (3.3%), C18:1 + C18:2 + iso-C18 + anteiso-C18 (54.8%)	17 cadavers	M: 49–68 y F: 2 wks−85 y	8 M 9 F	Reinertson et al. (12)
				9 normal human	23–52 y	M	
		Phospholipids	PC (28.00%), PA (3.36%), Eplas (11.49%), PE (6.97%), PS (9.49%), LPC (3.08%), PI (5.31%), AAPC (11.17%), SM (11.22%), DHSM (9.76%), CL (4.13%)	7	nd	nd	Meneses et al. (23)
		Sterols	Cholest-7-ene-3β-01 ester (nd)	2	nd	nd	Gray and Yardley (21)
		TAG DAG MAG	nd	nd	nd	nd	Nicolaides (24)
		Glycosphingolipids	nd	nd	nd	nd	Gray and Yardley (21)
Dermis		Ceramides	Cer [NS] (53.4%), Cer [NDS] (21.2%), Cer [NH] (7.3%). Cer [NP] (8%), Cer [AS] (3.4%), Cer [ADS] (1.1%), Cer [AH] (2.1%), Cer [AP] (3.5%)	4	33–47 y	F	Kendall et al. (19)
		FA of prostanoids	C20:3 n-6 (8.0%), C20:4 n-6 (90.5%), C20:5 n-3 (1.6%)	8	28–56 y	F	Kendall et al. (22)
		Hydroxy FA	C18:2 n-6 (50.3%), C20:3 n-6 (5.9%), C20:4 n-6 (40.9%), C20:5 n-3 (3.0%)	8	28–56 y	F	Kendall et al. (22)
		FA of N-acylethanolamides	C16:0 (38.7%), C18:0 (11.6%), C18:1 n-9 (18.3%), C18:2 n-6 (6.2%), C18:3 n-3 (1.1%), C20:4 n-6 (8.3%), C20:5 n-3 (4.1%), C22:6 n-3 (11.7%)	8	28–56 y	F	Kendall et al. (22)
		Total FA	C16:0 (19.9%), C18:0 (2.9%), C18:1 n-9 (44.8%), C18:2 n-6 (10.7%), C18:3 n-3 (0.7%), C20:4 n-6 (0.7%), C20:5 n-3 (0.1%), C22:6 n-3 (0.2%)	8	28–56 y	F	Kendall et al. (22)
		Phospholipids	PC (37.09%), PA (2.03%), Eplas (9.83%), PE (6.10%), PS (8.82%), LPC (5.53%), PI (5.17%), AAPC (6.56%), SM (15.86%), DHSM (4.58%), CL (2.04%)	7	nd	nd	Meneses et al. (23)
		TAG DAG	nd	nd	nd	F	Sjövall (25)
		Cholesterol esters	nd	nd	nd	nd	Kendall et al. (26)
Hypodermis		TAG	nd	nd	nd	nd	Kanitakis (3)
		FFA	nd	nd	nd	nd	Kanitakis (3)
		Total FA in hypodermis from mid-abdominal and mid-scapular	C10:0 (0.2%), C12:0 (0.6%), C14:0 (3.1%), C14:1 + iso-C14 + anteiso-C14 (0.5%), C16:0 (24.4%), C16:1 + iso-C16 + anteiso-C16 (9.2%), C18:0 (8.9%), C18:1 + C18:2 + iso-C18 + anteiso-C18 (53.8%)	17 cadavers	M: 49–68 y F: 2 wks−85 y	8 M 9 F	Reinertson et al. (12)
				9 normal human	23–52 y	M	

* *Classes present throughout the epidermis*.

** *No data available on concentrations of all these lipids*.

a *Protein-bound ceramide*.

b *EpiSkin human reconstructed epidermis model ceramide*.

c *Values calculated on the basis of the data in the publication*.

*TAG, triacylglycerol; DAG, diacylglycerol; MAG, monoacylglycerol; Cer, ceramide; FFA, free fatty acid; FA, fatty acid; PC, phosphatidylcholine; PA, phosphatidic acid; Eplas, ethanolamine plasmalogen; PE, phosphatidylethanolamine; PS, phosphatidylserine; LPC, lysophosphatidylcholine; PI, phosphatidylinositol; AAPC, alkylacylglycerophosphocholine; SM, sphingomyelin; DHSM, dihydrosphingomyelin; long-chain bases: DS, dihydrosphingosine; S, sphingosine; P, phytosphingosine; H, 6-hydroxy sphingosine; SD, 4,14-sphingaidene; fatty acid: N, non-hydroxy FA; A, alfa-hydroxy FA; [B], beta-hydroxy FA; [O], ω-hydroxy FA; [EO], esterified ω-hydroxy FA; [P-O], protein-bound FA; [1-O-E], 1-O-acylceramides with three hydrophobic chains; the third chain ester-linked to the primary hydroxyl in position 1 of the sphingoid base; [1-O-E(EO)], ceramides contain an ultra long-chain esterified with a linoleic acid in the N- position and a long to very long acyl chains in the 1-O- position of the sphingoid base; wks, weeks; y, years; M, male; F, female; nd, no data*.

The epidermis consists of 4 layers; counting from the bottom layer: stratum basale (SB), stratum spinosum (SS), stratum granulosum (SG), and stratum corneum (SC) (1). In the skin of the palms and soles, between the SG and SC, there is an additional layer—stratum lucidum (SL) (1). The SB consists mainly of a single layer of cuboidal basal cells, from which epidermal keratinocytes develop. The SB is constantly undergoing cell division. Therefore, old cells are pushed toward higher layers of the epidermis. In the SB, 45% of all lipids are polar, e.g., phosphatidylethanolamine (PE), phosphatidylcholine (PC), phosphatidylserine (PS), sphingomyelin (SM), and lysolecithin (LYS). Trace amounts of sphingolipids, which increase in the higher layers of the epidermis, can also be found (27). The main functions of the SB are proliferation, repair following damage to the epidermis, the reception of stimuli, and the synthesis of vitamin D. The SS is located between the SB and SG, and consists of 8–10 cell layers (1, 28). Keratinocytes are polygonal in shape with large, round nuclei. They are connected to each other by desmosomes so that they adhere more tightly to each other. As the cells migrate away from the SB, they begin to flatten. At the border of the SS and SG, lamellar bodies (LBs) begin to form (29). Involucrin production also begins, and there is an increase in the production of keratin 1 and keratin 10, which are markers of this layer (28). The SB and SS are where the synthesis takes place of cholesterol sulfate, which is a fraction of cholesterol substituted by a sulfoxy group at position 3 (30). The next layer, the SG, is composed of 3–5 layers of spindle-shaped cells with flattened nuclei (1). The cells in this layer contain keratohyalin granules with profilaggrin and loricrin. Profilaggrin is a precursor of filaggrin, involved in the binding of keratin fibers. The products of its degradation are counted among natural moisturizing factors (31). As a result of keratinization, granular cells remove all organelles and transform into corneocytes—dead cells of the epidermis (3). At the same time, there is an increase in the number of LBs, which at the boundary between the SG and SC, by exocytosis, caused by the increasing concentration of Ca^2+^ ions, secrete lipids and some hydrolytic enzymes which, in the intercellular space, form the intercellular lipid matrix (ICL) (2, 32). In the SG a decrease in polar lipids is observed and an increase in sphingolipid levels (Table 1) (8). Furthermore, there are the highest concentrations of cholesterol sulfate, which plays an important role in the process of epidermis exfoliation as it inhibits the proteases involved (30). In addition, the stabilization of lipid organization by dissolving cholesterol in the lamellar phases is also important (33). The SL is the intermediate layer between the SG and SC. It can be seen in certain regions of hairless skin. The keratinocytes in this layer are dead—it is considered the first dead layer of the epidermis. It contains lipid-rich protein, which makes it transparent and provides a barrier against water loss (1). The SC is the outermost layer of the epidermis and consists of 15–30 layers of cells—corneocytes.

The lipid bilayer of the cell membrane is converted into a single layer of acylceramides which are cross-linked with cornified envelope (CE) proteins (34). The membrane structure containing CERs bound to proteins is called the corneocyte lipid envelope (CLE) and serves to connect corneocytes to lipid sheets. The structure of the SC can be represented by the “bricks and mortar” model. The bricks are corneocytes immersed in the ICL, which plays the role of cement. The LBs at the interface release lipids to form lipid lamellae. The main ceramide precursors in lipid lamellae are glucosylceramides and SM. They are converted to CERs by β-glucocerebrosidase and sphingomyelinase when released into the extracellular space (35, 36). The SC is crucial for mechanical and biological protection and prevents excessive water evaporation.

The greatest quantities of lipids within the epidermis are cholesterol, FFAs and CERs. Cholesterol makes up 25% of the epidermal lipids. A major source of cholesterol in the skin is endogenous synthesis in this organ. Its main function is to improve the plasticity and rigidity of the membrane (37). It plays an important role in epidermal homeostasis, hence any change in its amount results in impaired barrier function and impaired epidermal exfoliation (38). Increased cholesterol synthesis occurs during permeability barrier repair (39). Sphingolipids are complex lipids with long-chain bases (LCBs) as their basic element. Most LCBs from sphingolipids have 12–22 carbon atoms with aliphatic amines that have two or three hydroxyl groups. Sphingolipids include CERs, glycosphingolipids, SM and sphingosine 1-phosphate, among others. Sphingolipids are involved in the formation of lipid microdomains and lipid rafts in biological membranes (40), the maintenance and stabilization of the nervous system (41), spermatogenesis (42), and play a role in apoptosis, signaling and proliferation (43). CERs play an important role in the formation and maintenance of the skin barrier (35, 36, 42, 44).

CERs are composed of LCBs and FAs varying in carbon chain length, degree of unsaturation, and position and number of hydroxyl group (45). LCBs have six sphingoid bases: sphingosine (S), 6-hydroxysphingosine (H), dihydrosphingosine (DS), phytosphingosine (P), dihydroxysphinganine (T) (46), and sphinga-4,14-diene (SD) (11). We can also distinguish five types of fatty acids that build ceramides: α-hydroxy fatty acids (A), non-hydroxy fatty acids (N), ω-hydroxy fatty acids (O) (46), and β-hydroxy fatty acids (B) (11). CERs esterified with additional FAs are preceded by the letter E before the base and the FA chain (46). There are 22 free ceramide classes and five protein-bound ceramides in the human epidermis (11, 14) (Tables 1, 2). EOS, EODS, EOH, EOP, and EOSD are the group of acylceramides. Some acylceramides are metabolized into protein-bound ceramides comprising one of the five LCBs and a P-O FA (34). CERs are an essential element in skin homeostasis. Changes in the composition or length of the FA chains that make up CERs can cause severe damage to the epidermal barrier or even lead to death. Acylceramide is essential for maintaining the proper packing of lipid lamellae (10, 48). CERs are involved in epidermal barrier renewal—their synthesis increases with keratinocyte differentiation (49). An excess of CERs leads to an increase in uncontrolled cell death and inflammation (50).

**Table 2:** Nomenclature for 22 free ceramide classes and 5 protein bound ceramide classes in human dermis and epidermis.

**Fatty acids**		**Non-hydroxy fatty acid [N]**	**A-hydroxy fatty acid [A]**	**ω-hydroxy fatty acid [O]**	**Esterified ω-hydroxy fatty acid [EO]**	**B-hydroxy fatty acid [B]**	**Protein-bound [P-O]**
**Amino base**							
	Sphingosine [S]	NS	AS	OS	EOS	BS	P-OS
	Phytosphingosine [P]	NP	AP	OP	EOP		P-OP
	6-hydroxysphingosine [H]	NH	AH	OH	EOH		P-OH
	Dihydrosphingosine [DS]	NDS	ADS	ODS	EODS		P-ODS
	4,14-Sphingaidene [SD]	NSD	ASD	OSD	EOSD		P-OSD
	Dihydroxysphinganine [T]	NT					

*Each ceramide class is represented by a combination of the abbreviations corresponding to its FA and amino base structure (11, 14, 47). [NS], combination of non-hydroxy FA (N) and sphingosine (S); [NP], combination of non-hydroxy FA (N) and phytosphingosine (P); [NH], combination of non-hydroxy FA (N) and 6-hydroxysphingosine (H); [NDS], combination of non-hydroxy FA (N) and dihydrosphingosine (DS); [NSD], combination of non-hydroxy FA (N) and 4,14-sphingaidene (SD); [NT], combination of non-hydroxy FA (N) and dihydroxysphinganine (T); [AS], combination of α-hydroxy FA (A) and sphingosine (S); [AP], combination of α-hydroxy FA (A) and phytoshingosine (P); [AH], combination of α-hydroxy FA (A) and 6-hydroxysphingosine (H); [ADS], combination of α-hydroxy FA (A) and dihydrosphingosine (DS); [ASD], combination of α-hydroxy FA (A) and 4,14-sphingaidene (SD); [OS], combination of ω-hydroxy FA (O) and sphingosine (S); [OP], combination of ω-hydroxy FA (O) and phytosphingosine (P); [OH], combination of ω-hydroxy FA (O) and 6-hydroxysphingosine (H); [ODS], combination of ω-hydroxy FA (O) and dihydrosphingosine (DS); [OSD], combination of ω-hydroxy FA (O) and 4,14-sphingaidene (SD); [EOS], combination of esterified ω-hydroxy FA (EO) and sphingosine (S); [EOP], combination of esterified ω-hydroxy FA (EO) and phytosphingosine (P); [EOH], combination of esterified ω-hydroxy FA (EO) and 6-hydroxysphingosine (H); [EODS], combination of esterified ω-hydroxy FA (EO) and dihydrosphingosine (DS); [EOSD], combination of esterified ω-hydroxy FA (EO) and 4,14-sphingaidene (SD); [BS], combination of β-hydroxy FA (B) and sphingosine (S); [P-OS], protein bound (P) combination of ω-hydroxy FA (O) and sphingosine (S); [P-OP], protein bound (P) combination of ω-hydroxy FA (O) and phytosphingosine (P); [P-OH], protein bound (P) combination of ω-hydroxy FA (O) and 6-hydroxysphingosine (H); [P-ODS], protein bound (P) combination of ω-hydroxy FA (O) and dihydrosphingosine (DS); [P-OSD], protein bound (P) combination of ω-hydroxy FA (O) and 4,14-sphingaidene (SD)*.

FAs, one of the components of ceramides, are a group of chemical compounds with a great deal of diversity, thus it is difficult to categorize them unequivocally. Table 3 presents the two most common divisions of acids: the first concerning the presence and number of double bonds and the length of the carbon chain in the molecule (Table 3).

**Table 3:** Classification of FAs according to carbon chain length and number of multiple bonds (5, 6).

Number of double bonds	Saturated Fatty Acid (SFA)
	Monounsaturated Fatty Acid (MUFA)
	Polyunsaturated Fatty Acid (PUFA)
Carbon chain length	Short chain fatty acid (SCFA) C2–C4
	Medium chain fatty acid (MCFA) C5–C11
	Long-chain fatty acid (LCFA) C12–C20
	Very long-chain fatty acid (VLCFA) C20–C25
	Ultra long-chain fatty acid (ULCFA) ≥ C26

**Figure 1:**
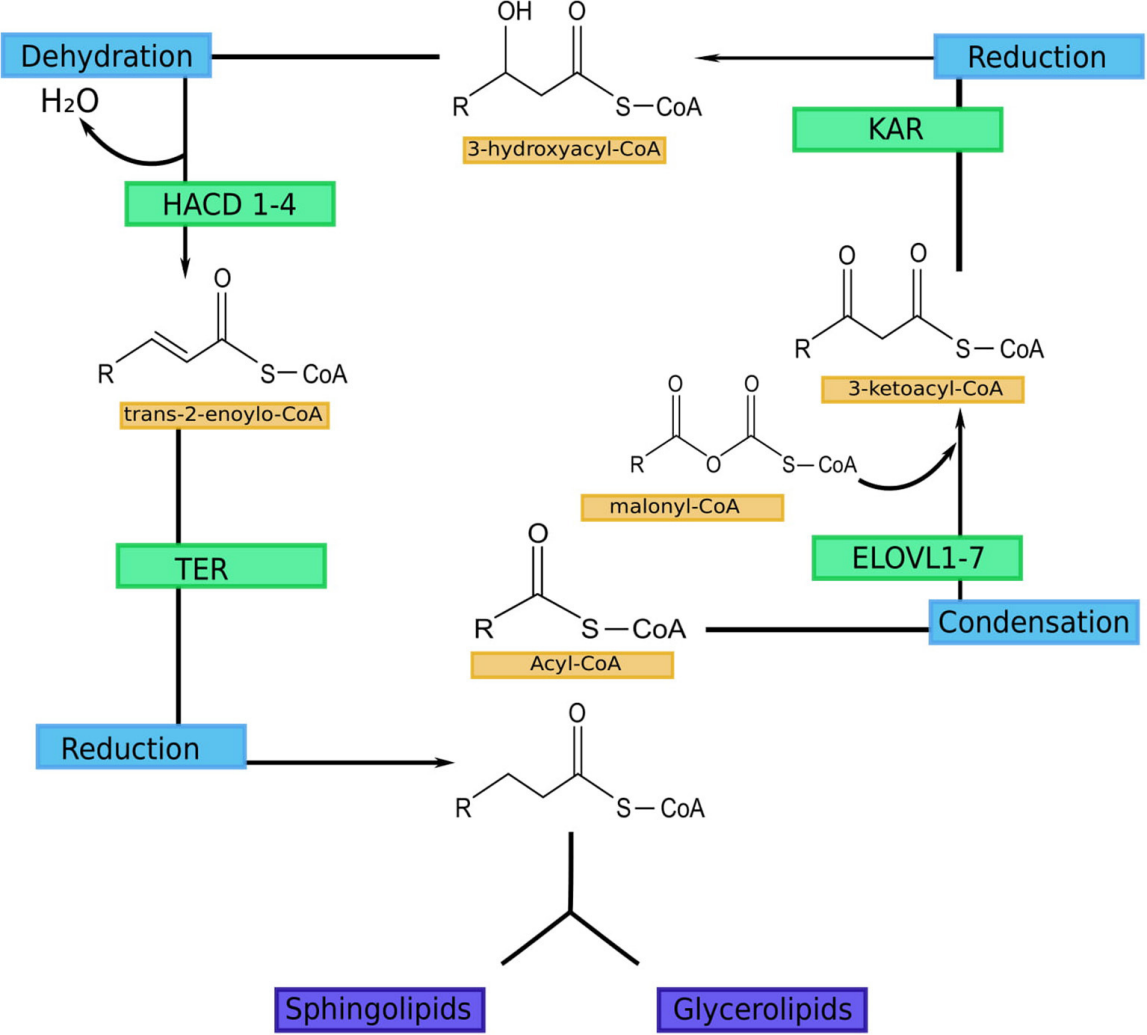
FA elongation cycle with involved enzymes in each step (5). ELOVL1-7-fatty acid elongase 1-7 (ELOVL1 elongates SFA with chain lengths of 18–24 carbons, ELOVL2 elongates PUFA with 20–22 carbon and SFA with 18–20 carbon chains; ELOVL3 elongates SFA with chain lengths of 18–24 carbons; ELOVL4 elongates long-chain PUFA and long-chain SFA of 24 carbon length to VLC-PUFA and VLC-SFA (≥26 carbons; ELOVL5 mediates elongation of long-chain PUFA and long-chain SFA between 18 and 22 carbons in length; ELOVL6 elongates SFA, MUFA and PUFA with 12–18 carbon chains; ELOVL7 elongates SFA with chain lengths of 18–22 carbons); KAR, 3-ketoacyl-CoA reductase; HACD 1-4, 3-hydroxyacyl-CoA dehydratase; TER, trans-2-enoyl-CoA reductase.

VLCFAs and ULCFAs are the most abundant group of FAs in the SC, but they are also present in the retina, meibomian gland, testis and brain. In addition, they can be found in the liver, lung, and kidneys (5, 6). The FA elongation process occurs in the endoplasmic reticulum and consists of four steps: elongation, reduction, dehydration, and reduction (Figure 1) (5, 6). Elongation is catalyzed by fatty acid elongase (ELOVL). Seven isoforms can be distinguished in mammals (ELOVL1-7) (51–53). This is a rate-limiting step. Reduction is catalyzed by 3-ketoacyl-CoA reductase (KAR), and NADPH is used as a cofactor (54). Dehydration is catalyzed by 3-hydroxyacyl-CoA dehydratase, which has 4 isoforms (HACD1-4). This is also a rate-limiting step (55, 56). The final step is also reduction, catalyzed by trans-2-enyl-CoA reductase (TER) (54). Each cycle results in the elongation of the carbon chain by two carbon atoms (51, 52).

VLCFAs and ULCFAs are degraded by beta-oxidation, and VLCFA-CoAs and ULCFA-CoAs are transported to peroxisomes where FA chains are converted to shorter acyl-CoAs. The resulting acyl-CoAs are then transported to the mitochondrial matrix where they undergo further steps of beta-oxidation (57).

### Very Long-Chain Fatty Acids and Ultra Long-Chain Fatty Acids in Skin Disorders

Considering the abundance of VLCFAs and ULCFAs, it is not surprising that there are plenty of enzymes and other proteins involved alongside them in multiple biochemical reactions and complex interactions. Disruption of these processes due to genetic defects in several genes encoding proteins enrolled in the metabolism of VLCFAs has clinical consequences leading, i.e., to inborn errors of metabolism and neurodegenerative disorders. Some also affect the skin and, in such cases, are referred to as genodermatoses, which are defined as inherited skin diseases. The majority of them are monogenic and can be inherited in an autosomal dominant, recessive or X-linked manner. The skin symptoms are common, but not exclusive, as several genodermatoses are multisystemic disorders. In fact, it is estimated that cutaneous findings can be present in around one third of all hereditary disorders (58). However, in the majority of them, the dysfunction of other organs is of principal clinical concern. Conversely, there are also genodermatoses with isolated skin symptoms only.

As expected, several proteins involved in the metabolism of ULCFAs are located in the epidermis and their mutations often result in an aberrant cornification process clinically manifested as isolated or syndromic ichthyosis or keratoderma. From the diagnostic perspective, the clinical features of those disorders, also referred to as Mendelian Disorders of Cornification, often overlap despite different molecular defects and, conversely, may be highly different even though the pathogenic variants occur in the same gene. Currently, several forms of autosomal recessive non-syndromic ichthyosis, including harlequin ichthyosis, lamellar ichthyosis, congenital ichthyosiform erythroderma and pleomorphic ichthyosis, are comprehensively named autosomal recessive congenital ichthyosis (ARCI). However, the clinical symptoms of ARCI may differ significantly between patients from a severe, even fatal phenotype to a mild outcome.

**Figure 2:**
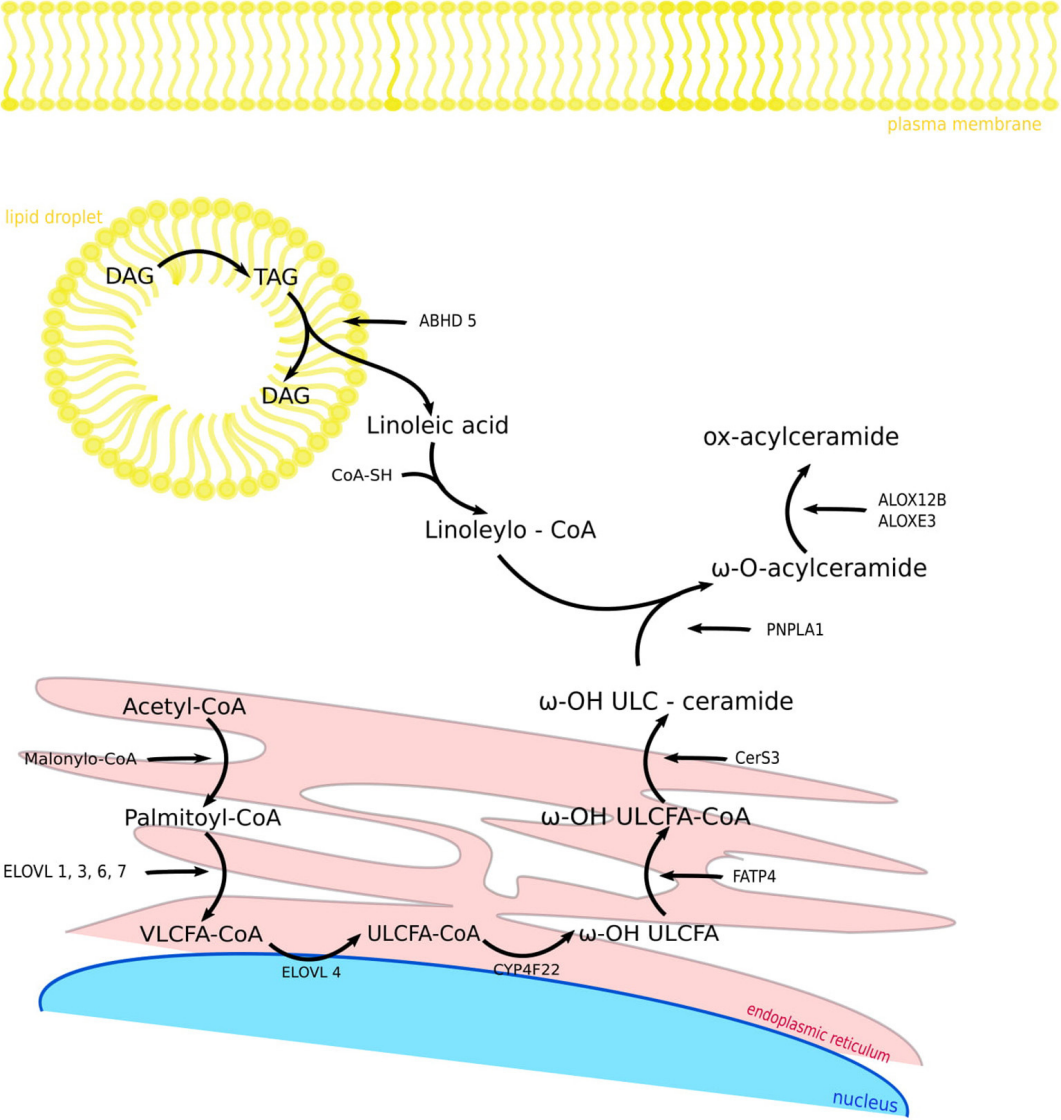
Components and biosynthetic steps of fatty acid modification in the skin. FASN1 catalyzes *de novo* synthesis of fatty acids from acetyl-CoA and malonyl-CoA. After activation to acyl-CoA esters, the acyl chain is elongated to VLCFA-CoA esters. CYP4F22 catalyzes ω-hydroxylation ULCFA-CoA to ω-OH ULCFA and FATP4 synthesize ω-OH ULCFA-CoA. CerS3 enables the synthesis of ω-OH ULC-ceramide. In the same time in lipid droplet TAG is hydrolyzed by ATGL activated by the ABHD5 to linoleic acid, which is synthesize with CoA-SH by receiving Linoleoyl-CoA. PNPLA1 catalyzes as a transacylase the formation of an ester bond between ω-hydroxyceramide and linoleoyl-CoA, so that we get ω-O-acylceramide. ALOX12B and ALOXE3 are responsible for oxidizing linoleic acid when it is attached to acylceramide. FASN1, fatty acid synthase 1; VLCFA-CoA, very long-chain fatty acid CoA; ELOVL, fatty acid elongase; ULCFA-CoA, ultra long-chain fatty acid CoA; CYP4F22, Cytochrome P450 Family 4 Subfamily F Member 22; FATP4, fatty acid transporter 4; CerS3, ceramide synthase 3; ABHD5, abhydrolase domain containing 5; PNPLA1, patatin like phospholipase domain containing 1; ALOX12B, arachidonate 12-lipoxygenase, 12R type; ALOXE3, arachidonate lipoxygenase 3; TAG, triacylglycerol; DAG, diacylglycerol.

Herein, we present an overview of selected proteins involved in the metabolism of VLCFAs and ULCFAs in the skin (Figure 2) with regard to recent findings connected with their functions and with skin pathology.

### Enzymes

ELOVL (elongases) 1-7 (3-ketoacyl-CoA synthases) are key enzymes involved in the elongation of saturated fatty acids (SFAs) and unsaturated FAs, which are essential for the proper functioning of several human systems and organs, the nervous system and the epidermis in particular. ELOVL1, 3 and 4 are enzymes involved in the first step of the elongation of SFAs and monounsaturated fatty acids (MUFAs) to VLCFAs. Each of these enzymes is expressed, among others, in the skin, therefore any disorder related to the mentioned elongases is manifested in the skin.

#### ELOVL1

Fatty acid elongase 1 (ELOVL1) is an enzyme involved in the cycle of VLCFA formation. It is involved in the first step in the preparation of acylceramide (59) and responsible for the elongation of saturated C18:0- to C26:0-CoA and monounsaturated C18:1- to C22:1-CoA (51, 53). Depending on its location in the epidermis, ELOVL1 interacts with ceramide synthases: ceramide synthase 2 (CERS2) and ceramide synthase 3 (CERS3). The cooperation of ELOVL1 with CERS2 takes place in the lower layers of the epidermis. ELOVL1 enables the formation of FAs C22:0 and C24:0, which are substrates in the process of obtaining CERs. In contrast, the coexpression of CERS3 takes place in the higher layers of the epidermis and this stimulates ELOVL1 to an additional cycle resulting in the formation of C26:0-CoA, which is further elongated by fatty acid elongase 4 (ELOVL4) (53, 60). The absence of VLCFAs C24:0 and C24:1 causes severe skin lesions. Recently, it was discovered that heterozygous mutations in the *ELOVL1* gene cause ichthyotic keratoderma, spasticity, hypomyelination, and dysmorphic facial features (IKSHD) disease. So far, only one mutation—p.Ser165Phe—was found to arise *de novo* in two unrelated patients of Polish origin (61, 62). Moreover, mice lacking *Elovl1* have an altered lipid lamellae structure, resulting in elevated TEWL (60). It has also been shown that ELOVL1 levels are decreased in psoriasis and atopic dermatitis (AD). In AD, interferon-γ (IFN-γ), which acts on keratinocytes to decrease the expression of the enzyme, is responsible for the downregulation of ELOVL1 (63, 64). In contrast, tumor necrosis factor α (TNF-α) and type 2 cytokines negatively affect the expression of the enzyme (65, 66).

#### ELOVL3

Fatty acid elongase 3 (ELOVL3) is involved in the elongation of saturated C16:0- to C22:0-CoA. It is expressed in brown adipose tissue and in the skin (51). In the *Elovl3*-ablate mice model, skin abnormalities can be observed due to a transient decrease in the ability to elongate saturated fatty acyl-CoAs during temporarily decreasing levels of C20:0 and C22:0 (67). The mice also have increased TEWL. The epidermal lipid composition is mildly altered with an increase in neutral lipids. It has also been shown that a lack of functional *Elovl3* in mice causes abnormalities in the SC—abnormal LBs and an abnormal membrane lipid composition. However, the lipid composition itself is not altered despite the altered phenotype (68).

Recently, another study on mice led to the discovery that the ELOVL3 enzyme is involved in the synthesis of C21:0 to C29:0 FAs, including odd and branched chains (69). Interestingly, according to the Human Gene Mutation Database (HGMD), only a single variant of mutation in *ELOVL3* was detected in humans so far. The variant occurred *de novo* and was detected in one child of a large cohort screened for molecular alterations causing autism spectrum disorders (70). A reduced expression of ELOVL3 by interleukin 4/interleukin 13 (IL-4/IL-13) was observed in a keratinocyte culture experiment—this resulted in an accumulation of FAs with shorter chains and a decrease in VLCFAs. A reduced expression of ELOVL3 by IL-4/IL-13 was observed in the SC of AD patients—this resulted in an accumulation of shorter chain FAs and a reduced level of VLCFAs. Moreover, after a siRNA-induced downregulation of ELOVL3/ELOVL6 expression in keratinocytes, the proportion of long-chain fatty acids (LCFAs) globally and in sphingolipids was decreased (71).

#### ELOVL4

Fatty acid elongase 4 (ELOVL4) is the enzyme responsible for elongating SFAs and polyunsaturated fatty acid (PUFA) ULCFAs-C26:0–C36:0 (51). It is the only elongase that extends the carbon chain beyond 26 carbon atoms. ELOVL4 catalyzes the first step in the preparation of acylceramides, which results in ULCFAs. Since the ULCFAs are components of skin CERs and glucosylceramides, they are essential in providing the hydrophobicity of lipid lamellae in the epidermis, and in the preservation of the water barrier (5, 6). Indeed, certain pathogenic variants occurring in the gene encoding ELOVL4 cause scaly and dry skin. These symptoms are caused by the absence of lamellar membranes in extracellular domains in the SC (72), which in turn are due to the lack of ω-O-acylceramides (72, 73). Similarly, ω-O-acylceramides are also involved in the lipid layer formation in the retina, acting against the evaporation of the aqueous tear film (74). In addition, besides the skin and retina, ELOVL4 is also expressed in the central nervous system and in the reproductive system (74).

The expression pattern of ELOVL4 explains, at least partially, the fact that skin syndromes are not isolated and, so far, were only identified as part of more systemic diseases, e.g., autosomal dominant spinocerebellar ataxia and erythrokeratodermia (75), and autosomal recessive syndromes, referred to as ichthyosis, intellectual disability and spastic quadriplegia (76) or neuro-ichthyotic disorder. Interestingly, among 21 different *ELOVL4* variants published so far, skin involvement was observed only in 8 cases. The majority of *ELOVL4* pathogenic variants led to autosomal dominant: Stargardt disease and spinocerebellar ataxia.

It has been proposed that the phenotype depends on the variant type and location within the gene: pathogenic variants leading to Stargardt disease and neuro-ichthyotic disorder, leading to protein truncation and the absence of the C-termination part, where the ER-retention motif is encoded. In the case of Stargardt disease, those variants tend to locate in exon 6. In spinocerebellar ataxia, missense variants are mainly detected and hence, although changed, still a full-length protein can be produced. The mechanism of Stargardt disease was investigated on a mice model. In transgenic mice expressing a pathogenic variant form of human *ELOVL4*:c.790_794delAACTT (p.Asn264Leufs^*^9), it was shown that an accumulation of undigested phagosomes and lipofuscin by the retinal pigment epithelium is followed by its atrophy and photoreceptor degeneration (77). Furthermore, Vasireddy et al. (72) observed on their mice model that heterozygous mice harboring a 5bp deletion in the *Elovl4* gene also had progressive photoreceptor degeneration, while in the case of homozygotes, severe skin symptoms were present and death occurred within the first few hours of life (72). This corresponds to severe ichthyosis, intellectual disability and spastic quadriplegia syndrome in humans.

Last but not least, recent studies on normal human cultured keratinocytes of AD and mice models show that IFN-γ significantly reduces ELOVL4, which may be one of the key findings explaining the mechanism of the chronicity of barrier function impairment in AD (63, 64).

#### CERS3

Ceramide synthase 3 (CERS3) is an enzyme expressed in the testis and skin (78, 79). This enzyme is responsible for the formation of epidermal-specific CERs and is one of the enzymes involved in the synthesis of acylceramides. Importantly, it is the only enzyme with the ability to synthetize ULC-ceramides (78–80). In the epidermis, the expression originates in the SB and increases with keratinocyte differentiation, so the highest amounts of CERS3 are present in the SG and SC. CERS3 cooperates with ELOVL1 and ELOVL4 (49, 60). In the lower epidermal layers, the cooperation of CERS3 and ELOVL1 catalyzes one more elongation cycle and produces C26:0-CoA, which can next be elongated by ELOVL4 (60). The coordinated expression of ELOVL4 and CERS3 is controlled by the peroxisome proliferator-activated receptor (PPAR) factor β/γ (49). CERS3 also has an ability to take over the functions of another ceramide synthase—CERS2 allowing uninterrupted ceramide synthesis (49). CERS3 deficiency results in decreased levels of acylceramides and ULC-CERs (≥C24 CERs) (78, 81), which cause skin barrier damage due to the impaired formation of intercellular lipid bilayers (82) and the decreased water permeability barrier (WPB) (35, 42).

Although *CerS3*-deficient mice had prominent skin symptoms and died shortly after birth (78), pathogenic variants of *CERS3* in humans are not lethal and the condition of human skin in affected people tends to improve with age. In 2013, the first cases of *CERS3* pathogenic variants in humans were reported (81, 83) and up to now, only 9 different pathogenic variants in this gene are known, according to the HGMD. They cause rare ARCI type 9, which is clinically characterized mainly by a collodion membrane at birth, generalized scaling with fine or large scales, and palmoplantar hyperlinearity. In some patients, large brownish scales on the lower extremities, acrogeria, ectropion, and alopecia may develop (84).

Along with studies on *ELOVL4* gene expression in the context of psoriasis and AD, the involvement of CERS 3 in the elucidation of the pathomechanisms of these disorders is also being investigated (63, 64).

#### CYP4F22

CYP4F22 is a protein belonging to the cytochrome P450 family 4. It is highly expressed in the epidermis, mainly in the SG (85). It is a fatty acid hydroxylase that catalyzes the ω-hydroxylation of ULCFAs (FAs >C26:0) (86, 87). In a mice *Cyp4f39e* knockout (KO) model (Cyp4f39e is a functional homolog of human CYP4F22), death occurred within 8 h of birth due to severe skin barrier disruptance. An increased thickness of corneocytes, and the presence of corneodesmosomes, which normally disappear in the upper layer of the SC, were observed. Miyamoto et al. (88) demonstrated these mice had reduced ω-OH CERs and they stored ULC-CERs. A significant decrease in acylceramide concentration was also observed (88).

The *CYP4F22* gene was discovered in 2006 (85) and subsequently, pathogenic variants were discovered in patients with ARCI. Around 55 pathogenic variants have been described since then, most of which are missenses. Recently, Nohara et al. (89) investigated CYP4F22 enzyme activity *in vitro* with several missenses and showed that the majority of them led to a marked reduction or loss of ω-hydroxylase activity. In two of the analyzed cases, however, the enzyme activity was comparable to the wild type (89). According to the authors, this could reflect the fact that either these mutations are not pathogenic or that patients with these variants have very mild ichthyosis symptoms. However, these were the results of *in vitro* studies, so the exact effect of those variants *in vivo* could be potentially different. The frequency of mutations in *CYP4F22* differs among the patient cohort and usually reaches 3–8% of ARCI patients (85, 90, 91). In one of the largest ARCI studies comprising 770 families, *CYP4F22* pathogenic variants were found in 54 families (87). The authors made an attempt to find genotype-phenotype correlations in their CYP4F22 cohort, but could not define any (87).

#### ABHD5

ABHD5 is an enzyme of the hydrolase family, also referred to as CGI-58, and also expressed in the epidermis. The enzyme activates adipose triglyceride lipase (ATGL, also known as PNPLA2) (92), thus providing fatty acids for the ω-O-esterification of CERs to yield acylceramides. Its expression increases during keratinization (93, 94). ABHD5 is involved in the derivation of linoleic acid necessary for the formation of acylceramides (95). Linoleic acid is required for acylceramide synthesis and CLE formation (94, 96). CLE abnormalities cause lethal, postnatal permeability barrier defect, which can be observed in *Abhd5* KO mice (95). Moreover, ABHD5 stimulates PNPLA1 in acylceramide synthesis. ABHD5 targets enzymes to lipid droplets, which facilitates the access of PNPLA1 to the required substrate (97, 98). Hence ABHD5 defects indirectly affect the energetic balance as well.

In humans, mutations in the *ABHD5* gene cause rare, multisystemic Dorfman-Chanarin syndrome (neutral lipid storage disease-NLSD) (99, 100). One of the characteristic (and diagnostic) features of this disease is the presence of ichthyosis and lipid droplets in granulocytes. It has been shown that ATGL inactivation, caused by molecular defects in ABHD5, leads to the accumulation of TAG-rich intracytoplasmic lipid droplets. ABHD5 is a co-activator of the hydrolase activity of ATGL. Lipid droplets can be observed in several tissues, which indeed reflects the multiorganic character of Dorfman-Chanarin syndrome, which includes, i.e., hepatomegaly and muscle weakness (99, 100).

#### PNPLA1

PNPLA1 represents a family of enzymes containing a patatin-like phospholipase domain (101). In the epidermis its expression occurs in the SG, and PNPLA1 localizes at the interface between the SG and SC layers (98, 102). It participates in O-acylceramide synthesis by catalyzing as a transacylase the formation of an ester bond between ω-hydroxyceramide and linoleate using triglyceride as the linoleate donor (98, 103, 104). PNPLA1 may be involved in the incorporation of ω-OH-Cer FAs as the last step in the production of acylceramides (105). PNPLA1 also plays an important role in keratinocyte differentiation (98). In *Pnpla1* KO mice, an accumulation of substrates required for acylceramide synthesis is observed: ω-OH CERs, ω-OH ULCFA. Consequently, there is excessive transepidermal dehydration. The proliferation of keratinocytes is also delayed. Furthermore, there is a lack of the corneocyte lipid envelope (CLE) associated with corneocytes (103). Mutations in the *Pnpla1* gene in mice also cause the abnormal secretion of compact lamellar granules at the SG and SC interface and the formation of lipid aggregates in corneocytes (98, 105). In addition, lipid lamellae have an abnormal alignment and the organization of intercorneocyte lipids is defective (105). Although PNPLA1 is known to localize on the cytoplasmatic lipid droplets, it has only recently been shown that in the case of mutations in *PNPLA1* genes, the accumulation of lipid droplets in fibroblasts is changed (106, 107). Indeed, mutations in the human *PNPLA1* gene are causative of ARCI (102, 105, 108–110). In patients with mutations in this gene, various skin symptoms occur, e.g., a collodion membrane at birth, erythroderma and ichthyosis; however, atopy and fungal infection tendency were also observed (111). Recent studies indicate an association between PNPLA1 single nucleotide polymorphism (SNP) rs4713956 and AD. The results suggest that the pathogenesis of AD may be due to a reduction in the combination of esterified ω-hydroxy FAs (EO) and sphingosine (S) (EOS) synthesis and insufficient CLE formation (112). Since the frequency of *PNPLA1* gene mutations among ARCI patients is rather low, there are no sufficient data yet to define a correlation between the genotype and the type of skin lesions (113).

#### ALOX12B and ALOXE3

2 (R)-lipoxygenase (12R-LOX) and lipoxygenase-3 (eLOX3) belong to the lipoxygenase family and are encoded by ALOX12B and ALOXE3, respectively. They act as dioxygenases in the epidermis (114, 115) and are responsible for oxidizing linoleic acids when they are attached to acylceramides (115, 116). In Alox12b and Aloxe3 KO mice, a decrease in CERs bound to cornified cell envelope (CCE) proteins was observed (115, 117, 118). Alox12b KO mice had a reduced amount of CERs with oxidized linoleic acid, which caused a loss of barrier function without alterations in proliferation, and the stratified organization of keratinocytes (118) and severe skin damage (118, 119). Mutations in ALOX12B and ALOXE3 genes in humans cause ARCI with generally a rather mild clinical manifestation, including erythema, scaling and mild palmoplantar keratoderma. According to a recent meta-analysis by Hotz et al. (120), in about 76 and 36% of patients with ALOX12B and ALOXE3 mutations, respectively, a collodion membrane was present at birth (120). In epidemiological studies, depending on the ethnicity, taken together, mutations in ALOX12B and ALOXE3 are detected in about 15–30% of ARCI patients (121). Moreover, in both genes, hot-spot mutations are known: p.(Pro630Leu) and p.(Arg234^*^) accounting for 61% of mutated ALOXE3 alleles and p.(Tyr521Cys) present in 22% of all ALOX12B mutated alleles (120).

#### PHYH

Phytanoyl-CoA hydroxylase (PHYH) is a peroxisomal enzyme involved in the α-oxidation of fatty acids, and converts phytanoyl-CoA to hydroxyphytanoyl-CoA (122, 123). PHYH deficiency in adults results in phytic acid (PA) accumulation (124), which leads to autosomal recessive Refsum disease. The symptoms of this disorder progress with life and include progressive retinitis pigmentosa and hearing loss, anosmia, polyneuropathy, cardiac arrhythmias, unsteadiness of gait, and ichthyosis (125). The symptom affecting the skin becomes apparent relatively late in life, as late as adolescence or even at the age of 30 or 40 years (126). The accumulation of PA in human skin causes an abnormal shape of lamellar bodies, which may cause a change in the organization of lipid lamellae (127). In addition, the complete loss of the CLE was described (127). Accumulated PA can replace linoleic acid in acylceramides, resulting in CLE atrophy (126).

#### FATP4

Fatty acid transporter 4 (FATP4) is a protein belonging to the membrane-bound FATP family and is encoded by SLC27A4 (128). The expression sites are the upper part of the SS and the SG (129–131). FATP4 is a major fatty acid CoA synthase for the production of ULCFAs by the synthesized ULCFA-CoA in the epidermis and can transport exogenous VLCFAs across the plasma membrane (128, 132–136).

FATP4 is predominant in the fetal epidermis, and is crucial for epidermal barrier formation in mice neonates, but is not important for the maintenance of this barrier in adult skin (130). In mutant mice the presence of severe skin barrier abnormalities causing increased TEWL is manifested by hyperkeratosis and acanthosis (129, 131, 137). Mice with *Fatp4* mutations have impaired lipid lamellae formation and keratinocyte differentiation (137). This is caused by decreased acyl chain ceramides ≥26C and increased ceramides ≤ 24C (129, 131), but also by increased levels of FFAs (137). All these changes in the amount and composition of FFAs result in changes in the organization of lipid lamellae, and increased TEWL (138).

FATP4 is encoded by the *SLC27A4* gene, the mutations of which lead to syndromic autosomal recessive ichthyosis prematurity syndrome (IPS), one of the disorders commonly referred to as ARCI (132). IPS is characterized by premature birth, respiratory distress, skin abnormalities at birth, and eosinophilia (139). Although the perinatal complications are life-threatening, the symptoms may alleviate with time (140). IPS is considered to be a rare disorder, being more frequent in Scandinavian countries, probably due to founder mutation (141, 142). However, up to now, 23 distinct pathogenic mutations have been reported worldwide (according to the HGMD) and some authors claim that the frequency of this disease is underestimated (143).

## Discussion

Lipids are important building blocks of the skin. Any changes in the amount and composition of lipids cause skin diseases. In this work we focus on VLCFAs and ULCFAs, and mutations in the genes responsible for the metabolism of these FAs. The small number of studies on VLCFAs and ULCFAs may be due to cognitive difficulties related to limitations in the choice of the research model. In most studies, the research model is mice, whose disease symptoms are more severe than in humans. Additionally, some mutations in humans are so rare that the exact pathomechanism of the disease has not yet been worked out. However, the development of research techniques and lipid analysis methods allows us to conclude that advances in the understanding of epidermal ceramide synthesis and metabolism, and especially acylceramides, will contribute to the development of effective, innovative therapies related to functional epidermal lipids in ichthyoses and ichthyosis syndromes.

## Data Availability Statement

The original contributions presented in the study are included in the article/Supplementary Material, further inquiries can be directed to the corresponding authors.

## Author Contributions

AM conceived and designed the review and verified the manuscript. AZ and KW-T studied the literature and wrote the manuscript. All authors accepted the final version of the review. All authors have read and agreed to the published version of the manuscript.

## Conflict of Interest

The authors declare that the research was conducted in the absence of any commercial or financial relationships that could be construed as a potential conflict of interest.

## Publisher's Note

All claims expressed in this article are solely those of the authors and do not necessarily represent those of their affiliated organizations, or those of the publisher, the editors and the reviewers. Any product that may be evaluated in this article, or claim that may be made by its manufacturer, is not guaranteed or endorsed by the publisher.
